# Engaging novel cell types, protein targets and efficacy biomarkers in the treatment of diabetic nephropathy

**DOI:** 10.3389/fphar.2014.00185

**Published:** 2014-08-07

**Authors:** Ryan M. Fryer, Carine M. Boustany-Kari, Scott M. MacDonnell

**Affiliations:** Boehringer Ingelheim Pharmaceuticals, Inc., Cardiometabolic Diseases ResearchRidgefield, CT, USA

**Keywords:** diabetic nephropathy, chronic kidney disease, angiopoietin-like protein 4, N-acetyl-seryl-aspartyl-lysyl-proline, mineralocorticoid antagonist, PF-03882845, ERK5, MEK5

Diabetic nephropathy (DN) is a well-known complication of diabetes and leading cause of chronic renal failure and end-stage renal disease (ESRD). Currently, angiotensin converting enzyme inhibitors (ACE-I) and angiotensin receptor blockers (ARBs) are the standard of care for this disease although trials targeting the renin-angiotensin-aldosterone system demonstrate that they delay the progression to ESRD to only a limited extent [~18% relative risk reduction (Brenner et al., 2001; Lewis et al., 2001)]. Furthermore, dual-targeting of this pathway appears less-than-promising, if not deleterious, as evidenced by the failure of the recent ALTITUDE trial with the direct renin inhibitor, aliskiren, where in combination with losartan, therapy reduced albuminuria but failed to impact glomerular filtration rate (Parving et al., 2012). Thus, limited efficacy coupled with our incomplete understanding of this multifaceted disease highlights the importance of unraveling novel targets based on a greater understanding of the pathogenesis of renal impairment in diabetes. We must look beyond ACE-Is/ARBs and capitalize on blending new technologies, target-disease associations, physiology-based insight, and our enhanced ability to mine vast quantities of literature and data as a means to identify new targets and then deliver novel drug candidates into the clinic.

Various signaling pathways encompassing inflammation, fibrosis, and oxidative stress are implicated in the pathogenesis of DN (Reidy et al., 2014) and perhaps the key to novel and efficacious therapies lays within the identification of druggable targets that encompass these diverse processes. Can a population of inflammatory cells be targeted to slow disease progression? Or, should we look more closely to podocyte-selective targets central to the regulation of glomerular filtration? Alternatively, do we set our sights within the complicated environment of the renal microvasculature? While early DN may represent an overtly angiogenic environment, advanced disease likely represents a condition of capillary loss and excess anti-angiogenic activity (Advani and Gilbert, 2012). If so, can we reasonably target the renal microvasculature in a manner expected to yield benefit across various stages of DN or might this type of targeted therapy treat only a smaller subset of the DN population?

In this Research Topic, contributing authors explored four sub-areas within the realm of chronic kidney disease and DN including capitalizing on the discovery of novel mineralocorticoid receptors with potentially lesser risk for hyperkalemia (Orena et al., 2013), the potential for modulation of a secreted glycoprotein already recognized as a contributor to minimal change disease and now also as a target in DN (Chugh et al., 2014), use of an endogenous anti-fibrotic peptide in renal disease (Kanasaki et al., 2014), and basic science that investigated the expression of mitogen-activated protein kinases (MAPKs) in podocytes as potential new targets in DN (Badshah et al., 2014), an interesting area that may offer another pathway and novel players for targeted therapies in this disease.

Indeed, results reported in this Research Topic by Orena et al. (2013), who investigated renal protection against aldosterone-mediated renal disease, in uninephrectomzied rats on high salt, demonstrate the anti-proteinuric and renal anti-fibrotic effect of PF-03882845 with a reduced risk of hyperkalemia compared to traditional mineralocorticoid (MR) antagonist. Should these findings translate to a greater safety profile in humans, this therapy might constitute a significant and beneficial add-on to the current standard of care.

Chugh et al. (2014) describe, in detail, the discovery of angiopoietin-like protein 4 (ANGPTL4), secreted by podocytes, as a major player in human nephrotic syndrome and highlight the strategy used to identify and then selectively investigate genes and proteins that lay at the intersection of proteinuria, hyperlipidemia, and edema during disease progression. They suggest that sialylation-based, and recombinant mutated ANGPTL4-based, therapeutics could hold promise in the treatment of common forms of proteinuric kidney disease including DN. Moreover, it could be envisioned that others could capitalize on a strategy similar to that employed by the authors to identify additional novel genes and proteins that could be targeted for the treatment of human DN.

Investigating endogenous molecules as potential means for identification of novel therapeutics, Kanasaki et al. (2014) discuss novel therapeutic possibilities for renal disease and detail work surrounding N-acetyl-seryl-aspartyl-lysyl-proline (AcSDKP), a substrate for ACE and theoretical candidate for anti-fibrotic therapeutics.

A large body of published data suggests that podocytes play a major role in the development of proteinuria and kidney disease by their ability to regulate glomerular filtration. Additionally, analysis using samples from both type I and II diabetic patients indicated that podocyte number is highly correlated with proteinuria and appears to be one of the best disease predictors (White et al., 2002). However, the mechanisms of podocyte injury leading to foot process effacement and proteinuria are unclear. In the current research topic, Badshah et al. (2014) investigated the role of atypical MAP kinase Erk5 in mediating TGFβ1-induced podocyte damage. Indeed, the role of ERK5 in the proliferation, apoptosis, and motility of cultured human podocytes was explored using BIX02188, a novel Mek5 inhibitor that was experimentally shown to reduce TGFβ1-induced Erk5 phosphorylation. They confirmed the presence of three splice variants of Erk5 in human podocytes that were phosphorylated by Mek5 in a TGFβ1-dependent manner. TGFβ1-induced the proliferation and apoptosis of podocytes while decreasing motility. While proliferation was reduced by inhibiting Erk5 phosphorylation with BIX02188, no changes in apoptosis or motility were observed. Most importantly, inhibition of Mek5 with BIX02188 and subsequent loss of Erk5 phosphorylation, reduced TGFβ1-induced alterations of cellular phenotype, as determined by a reduction in the expression of P-cadherin and increased α-SMA, in addition to a reversal of TGFβ1-indcued loss of barrier function. These results describe for the first time the expression of Erk5 in podocytes and identify it as a potential target for the treatment of diabetic renal disease.

Taken together, the findings highlighted in this research topic provide novel insight into new or pre-existing pathways implicated in disease. As researchers continue to seek and investigate mechanisms causal of disease, the importance of biomarkers predictors of efficacy should be emphasized. Novel biomarkers tailored toward the targeted pathways (such as inflammation, fibrosis, and endothelial function) and linked to disease are urgently needed to provide clear efficacy signals in phase II clinical trials and minimize further failures in phase III.

## Conflict of interest statement

The authors were Editors of the Research Topic, and were employed by Boehringer Ingelheim Pharmaceuticals, Inc. at the time of publication. Dr. Carine M. Boustany-Kari was an author in the publication by S. Orena et al. published in this Research Topic.
